# A technique for repairing rotator cuff transtendinous tears with a remnant attached to the footprint

**DOI:** 10.1186/s13018-021-02449-8

**Published:** 2021-05-03

**Authors:** Hyung-Suk Choi, Byung-Ill Lee, Jae-Hyung Kim, Hyung-Ki Cho, Gi-Won Seo

**Affiliations:** 1 Department of Orthopaedic Surgery, Soonchunhyang University Hospital Seoul, 59, Daesagwan-ro, Seoul, 04401 South Korea; 2 Department of Orthopaedic Surgery, Soonchunhyang University Hospital Gumi, 179, 1gongdan-ro, Gumi, Gyeongsangbuk-do 39371 South Korea

## Abstract

**Background:**

Some unusual rotator cuff (RC) tears are located in more proximal tendinous portions, with substantial remnant tissue attached to the footprint. The two options for surgical repair are sacrificing or preserving the remnant tissue. We introduce a surgical repair technique that preserves as much of the remnant footprint as possible.

**Surgical technique:**

A double-loaded suture anchor is inserted into the subchondral bone at the medial portion of the RC footprint; the lateral remnant tissue is preserved. Each strand is shuttled and repassed through the medial portion of the tendon in a mattress fashion using a suture hook device. Then, multiple no. 1 PDS sutures are passed through the medial and lateral stumps and left untied. Strands from the suture anchor are first tied in a double mattress fashion. Then, the repair is completed by tying the remaining no. 1 PDS sutures.

**Conclusions:**

We propose a remnant-preserving RC repair technique for transtendinous RC tears with sufficient tissue remaining within the RC footprint. This technique appears advantageous in terms of re-establishing an environment that promotes tendon healing after repair.

## Introduction

**Fig. 1:**
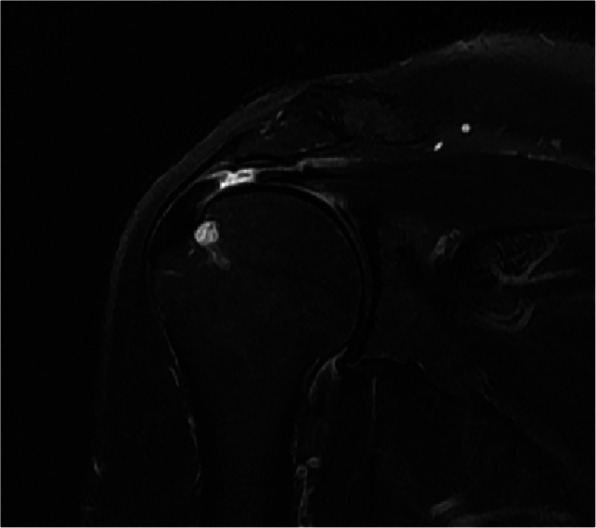
Preoperative T2-weighted fat-saturated magnetic resonance image showing a transtendinous rotator cuff tear with substantial remnant tissue attached to the footprint

Rotator cuff (RC) tears are commonly encountered in the orthopedic department [1, 2]. Although the pathogenic mechanisms of RC tears are still debated [3], when a tear occurs, the RC tendon is usually detached from its footprint on the greater tuberosity of the proximal humerus [4]. Thus, RC repair has been developed from a single row anchor repair technique to a transosseous-equivalent technique to improve tendon-to-bone healing [5]. In some cases, however, the tear is located more proximal to the footprint, with substantial distal remnant tissue attached to the greater tuberosity (Fig. 1). Because the RC footprint within the greater tuberosity is hidden by the remnant distal sump, sacrificing or preserving the tissue is a controversial decision.

If the remnant tissue is sacrificed, a suture anchor can be placed accurately in the footprint under direct visualization, which may effectively restore the proximally retracted portion of the RC to its original location. This might, however, reduce footprint coverage by sacrificing the distal stump. The rotator cuff tendon has many mechanoreceptors that contribute to shoulder proprioception [6, 7]. Gumina et al. found that the bigger the RC tear, the more shoulder proprioception is altered [8]. Therefore, it is reasonable to assume that sacrificing the remnant tissue may also sacrifice mechanoreceptors, ultimately affecting postoperative proprioception.

Therefore, as much remnant tissue as possible should be preserved in tears proximal to the RC footprint. Similarly, preservation of the remnant tissue has recently attracted attention in anterior cruciate ligament (ACL) reconstruction [9, 10]. Besides retaining proprioceptive mechanoreceptors, preserving the remnant tissue is advantageous for enhancing revascularization and improving joint stability [11]. Therefore, we hypothesized that, as in ACL reconstruction [12], retaining the remnant RC tissue in RC repair promotes satisfactory functional outcomes after repair surgery.

In this technical note, we describe an arthroscopic remnant-preserving repair technique for RC tears with substantial remnant tissue in the footprint.

## Surgical technique

Under general anesthesia, the patient is placed in a lateral decubitus position. Lateral arm traction of approximately 4.5 kg is applied using a traction device. Generally, three to five arthroscopic portals are used to perform the surgery (posterior, posterolateral, lateral, anterior, and anterolateral). Diagnostic arthroscopy of the glenohumeral joint and subacromial space is performed, along with adequate debridement of the reactive synovitis or bursa. Acromioplasty is performed if there is a huge bony spur. Then, the location and characteristics of the RC tear are evaluated carefully.

**Fig. 2:**
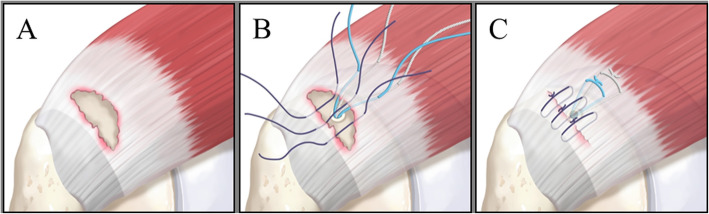
Illustration of a remnant-preserving rotator cuff repair. **a** Transtendinous rotator cuff tear with substantial remnant tissue attached to the footprint. **b** In most cases, the tear is repaired with simple sutures; three sutures are passed through the medial and lateral stumps. When the remnant tissue quality is poor or there is over-tension of the medial stump, a suture anchor is added with strands passed through the medial stump in a mattress fashion. **c** Repaired tendon with minimal tension

After the tear edges have been debrided, we punch a hole in the medial side of the footprint through an accessory superolateral portal. Subsequently, double-loaded suture anchors (5.5 mm, Bio-Corkscrew FT Suture anchor, Arthrex, Munich, Germany) are inserted through the accessory superolateral portal and fixed to the hole. Each strand is shuttled and re-passed through the medial portion of the tendon in a mattress fashion, using a suture hook device. The number of suture anchors varies with the size of the tear. Then, multiple no. 1 PDS sutures are passed through the medial and lateral stumps and left untied. Laterally mobilizing the medial stump with the grasping device, strands from the suture anchor are first tied in a double mattress fashion. Then, the repair is completed by tying the remaining no. 1 PDS sutures (Fig. 2).

## Postoperative rehabilitation

The shoulders were immobilized for 3 weeks using a sling immobilizer with an abduction pillow. Passive and assisted active exercises were initiated for forward flexion and external rotation, avoiding provocation of pain. After 6 weeks, patients began strengthening exercises of the RC and scapular stabilizers. Full return to sports and heavy labor were allowed after 6 months depending on individual functional recovery.

## Discussion

**Fig. 3:**
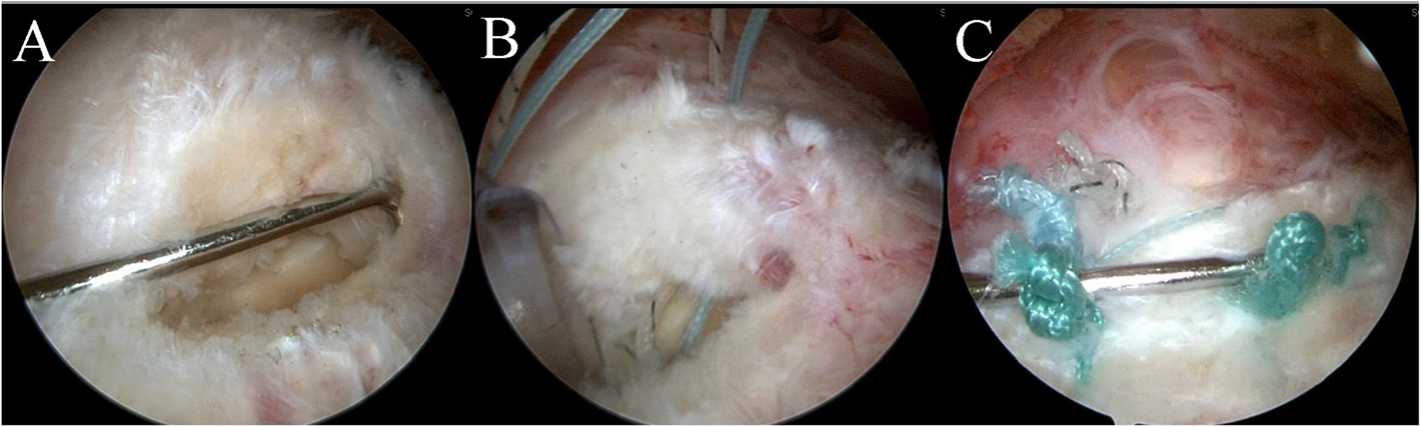
**a** Lateral view of a transtendinous rotator cuff tear with substantial tissue attached to the footprint. **b** Strands from a suture anchor passed through the medial stump in a mattress fashion. **c** Repaired rotator cuff with minimal tension

The aim of RC repair is to restore the anatomical location and mechanical performance of the RC sufficiently to withstand the loading associated with functional activity [13]. However, besides restoring its mechanical stability, enabling RC tendon healing under ideal conditions, to re-establish its biomechanical properties, plays an important role in successful RC repair [14]. Therefore, we postulated that preserving the remnant RC tissue in a transtendinous RC tear would be better than sacrificing the tissue for the following reasons (Fig. 3).

First, preserving the remnant tissue may help maintain proprioception of the RC. Joint structures are innervated by mechanically sensitive receptors termed mechanoreceptors that relay information to the central nervous system regarding movement, position, and forces exerted on shoulder structures [7, 15–19]. In the shoulder joint, there are mechanoreceptors in the coracoacromial ligament, RC tendons, musculotendinous junctions of the RC, and joint capsule [16]. It is reasonable to speculate that RC tears are associated with structural and functional alterations of proprioceptors [18–23]. Reduced or inconsistent proprioceptive information from the injured muscle–tendon unit and altered muscle reflex activity may impair shoulder proprioception and contribute to impaired kinematics and muscle recruitment. Consequently, the preserved remnant tissue on the footprint may help maintain as many mechanoreceptors as possible.

Second, the blood flow within the RC would also be preserved. The posterior humeral circumflex artery is the main vessel supplying blood to the supra- and infraspinatus tendons [24]. Because the posterior humeral circumflex artery provides blood to the RC flowing from the humeral attachment toward the proximal RC, the remnant within the footprint has high vascularity in a transtendinous RC tear [25]. As a result, the RC blood supply would inevitably be compromised if the remnant tissue were sacrificed.

Third, the natural enthesis of RC is maintained. The normal anatomy of the RC has four zones, from tendon to fibrocartilage to calcified fibrocartilage to bone. The ultimate goal of surgical repair is to replicate as much of this native enthesis as possible [26]. Su et al. reported that enthesis-preserving RC repair gave better histological and biomechanical results than enthesis-removal RC repair in a rabbit model [27]. By preserving the footprint remnant tissue, the RC can be repaired effectively with its native enthesis preserved within the RC.

Considering these advantages, we recommend a remnant-preserving tendon-to-tendon repair for transtendinous RC tears. If there is concern about the tissue quality of the lateral tendon segment or over-tension of the medial tendon segment, a suture anchor can be added for a more secure repair and to prevent retraction when necessary. Using this technique, anatomical repair of transtendinous RC tears can be achieved without excessive tension, and a biomechanical condition that promotes satisfactory tendon healing is established owing to the preserved remnant tissue.

## Data Availability

Not applicable

## Abbreviations

RC: Rotator cuff
ACL: Anterior cruciate ligament
